# Identification of Compounds with Potential Therapeutic Uses from Sweet Pepper (*Capsicum annuum* L.) Fruits and Their Modulation by Nitric Oxide (NO)

**DOI:** 10.3390/ijms22094476

**Published:** 2021-04-25

**Authors:** Lucía Guevara, María Ángeles Domínguez-Anaya, Alba Ortigosa, Salvador González-Gordo, Caridad Díaz, Francisca Vicente, Francisco J. Corpas, José Pérez del Palacio, José M. Palma

**Affiliations:** 1 Group of Antioxidant, Free Radicals and Nitric Oxide in Biotechnology, Food and Agriculture, Department of Biochemistry, Cell and Molecular Biology of Plants, Estación Experimental del Zaidín, CSIC, 18008 Granada, Spain; luciaguevaramonge@gmail.com (L.G.); mardomana2191@gmail.com (M.Á.D.-A.); alba10081996@gmail.com (A.O.); salvador.gonzalez@eez.csic.es (S.G.-G.); javier.corpas@eez.csic.es (F.J.C.); 2 Department of Screening & Target Validation, Fundación MEDINA, 18016 Granada, Spain; caridad.diaz@medinaandalucia.es (C.D.); francisca.vicente@medinaandalucia.es (F.V.); jose.perezdelpalacio@medinaandalucia.es (J.P.d.P.)

**Keywords:** fruit ripening, gingerglycolipid A, HPLC-HRMS, melatonin, nitric oxide, phytosphingosin, quercetin, transcriptomics, L-tryptophan

## Abstract

Plant species are precursors of a wide variety of secondary metabolites that, besides being useful for themselves, can also be used by humans for their consumption and economic benefit. Pepper (*Capsicum annuum* L.) fruit is not only a common food and spice source, it also stands out for containing high amounts of antioxidants (such as vitamins C and A), polyphenols and capsaicinoids. Particular attention has been paid to capsaicin, whose anti-inflammatory, antiproliferative and analgesic activities have been reported in the literature. Due to the potential interest in pepper metabolites for human use, in this project, we carried out an investigation to identify new bioactive compounds of this crop. To achieve this, we applied a metabolomic approach, using an HPLC (high-performance liquid chromatography) separative technique coupled to metabolite identification by high resolution mass spectrometry (HRMS). After chromatographic analysis and data processing against metabolic databases, 12 differential bioactive compounds were identified in sweet pepper fruits, including quercetin and its derivatives, L-tryptophan, phytosphingosin, FAD, gingerglycolipid A, tetrahydropentoxylin, blumenol C glucoside, colnelenic acid and capsoside A. The abundance of these metabolites varied depending on the ripening stage of the fruits, either immature green or ripe red. We also studied the variation of these 12 metabolites upon treatment with exogenous nitric oxide (NO), a free radical gas involved in a good number of physiological processes in higher plants such as germination, growth, flowering, senescence, and fruit ripening, among others. Overall, it was found that the content of the analyzed metabolites depended on the ripening stage and on the presence of NO. The metabolic pattern followed by quercetin and its derivatives, as a consequence of the ripening stage and NO treatment, was also corroborated by transcriptomic analysis of genes involved in the synthesis of these compounds. This opens new research perspectives on the pepper fruit’s bioactive compounds with nutraceutical potentiality, where biotechnological strategies can be applied for optimizing the level of these beneficial compounds.

## 1. Introduction

Pepper fruit is one of the most consumed horticultural product worldwide. Pepper (*Capsicum annuum* L.) is an annual herbaceous plant originating from South America which belongs to the Solanaceae, a family that includes a great number of important crops for the human diet and of economic interest, such as tomato (*Lycopersicum esculentum*), potato (*Solanum tuberosum*), and aubergine (*Solanum melongena*) [1,2,3]. Solanaceae fruits display morphological diversity in size, shape, and color among both species and varieties within each species. The fruits’ color is the result of their chemical composition, determined by the proportion of pigments, for instance, carotenoids, chlorophyll, flavonoids, and anthocyanins, most of them taking part in the so-called secondary metabolism [3,4,5]. In fact, plant species are precursors of a wide variety of secondary metabolites that, besides having useful activity for themselves, can also be used by humans for their benefit. Regarding pepper fruits, a colorful assortment of varieties can be observed, such as red, yellow, orange, purple and others once the fruits have ripened. Besides their visual features, pepper fruits are also differentiated according to their organoleptic characteristics and culinary uses. Thus, hot and sweet pepper fruit varieties can be found with a huge number of local names [1,6,7,8]. The different ways of processing food allow peppers to be consumed raw or cooked, as a powder, as a spice, or as jarred food. This great versatility in terms of culinary uses and the considerable increase in production over the last years justifies the interest and economic importance of the culture of pepper.

Peppers are a good source of antioxidants vitamin C (ascorbic acid, ascorbate), vitamin A (β-carotene as a precursor) and diverse minerals. As a matter of fact, 50–80 g of pepper fruits are sufficient to provide 100% and 25% of the recommended dietary allowance (RDA) of vitamin C and A, respectively [6,7,8,9,10,11,12,13,14]. Vitamin C, which might be considered as the paradigm of the low molecular weight antioxidants, is essential in animals and humans since it participates in many metabolic pathways and in the prevention of a number of pathologies [12,13,14,15,16,17,18]. Vitamin A is obtained once β-carotene breaks down symmetrically, so each carotene molecule gives rise to two vitamin A molecules. Vitamin A is important in the regulation of redox homeostasis in diverse physiological events related to vision, growth and development, among others [18,19,20,21,22].

However, pepper fruits are also rich in other carotenoids like capsanthin, capsorubin and lutein. Capsanthin is basically present in mature pepper fruits where is the main carotenoid, and is responsible for the red color [1,13,23]. This pigment is known for its anticarcinogenic properties and the preventive effect against atherosclerosis and obesity [24,25]. Lutein is mostly present in unripened green fruits being the main carotenoid. This compound prevents age-associated macular degeneration, and reduces the risk of apoplexy, cardiovascular disorders and cancer [24,26,27]. Additionally, pepper fruits contain a series of bioactive compounds, whose content varies depending on the variety, the part of the fruit (either pericarp, placenta or seeds), the maturation stage, the cultivation system, the climate, and the processing and storage practices [1,13]. They include phenolic compounds, which display beneficial health effects based on the protection against damage produced by oxidizing agents. This group includes flavonoids such as isoflavones, anthocyanidins, chalcones, and anthocyanins. In general, flavonoids have antibacterial, antifungal, antioxidant, and anticancer effects [1,8,13,28].

Capsaicinoids are specialized metabolites produced exclusively by the Capsicum genus, responsible for the pungency of hot pepper fruits [7,13,29]. This is a family of about 23 compounds where capsaicin and dihydrocapsaicin are the two main chemicals accounting for about 95% of total capsaicinoids [6,13,30]. Special attention has been paid to capsaicin, which has been carefully studied for its analgesic, antioxidant, anti-inflammatory, and anti-obesity properties [31,32,33]. The proapoptotic activity of capsaicin is mediated by transient receptor potential vanilloid type-1 (TRPV1) in many types of cancers. Capsaicin has also been found to induce phosphorylation of the tumor suppressor protein p53, which leads to its activation [33,34,35,36,37]. Consequently, the metabolic and biochemical content of *Capsicum* species is not only valuable for the plant itself, but also for human health due to its potential use in diverse therapies according to the biological activity [1,13].

Additionally, it has been reported that the metabolomic profile of pepper fruits can be modified by applying certain procedures. Thus, it was demonstrated that treatment with exogenous nitric oxide (NO) not only delayed the ripening of pepper fruits, but it also promoted an increase of about 40% of the ascorbate content [14,38]. Ripening of pepper fruits is characterized by color shifts from immature green to ripe red, yellow, orange or purple according to the variety. During this event, among others, chlorophyll breakdown and synthesis of new carotenoids and anthocyanins, synthesis of new protein and cleavage of former ones, and cell wall softening occurs [6,7,14,39,40,41,42]. From a metabolic point of view, this physiological process is accompanied by an increase in the lipid peroxidation and the activity of some enzymes such as the superoxide-generating respiratory burst oxidative homolog (Rboh) and NADP-dehydrogenases, as well as by higher NADPH levels and some post-translational modification of proteins. Conversely, during ripening, NO content and catalase activity lower [43]. The content of the main non-enzymatic antioxidants (ascorbate, glutathione, carotenoids and polyphenols) has also been investigated in pepper fruit ripening [7,8,28,44,45,46,47,48]. NO treatment was shown to prevent some of the changes undergone by these parameters, thus confirming the delaying effect of the ripening process indicated above [38,49,50].

Traditional medicine is based on the use of known natural products and/or synthesized secondary metabolites [51]. The identification of the myriad of natural metabolites with beneficial health effects of plant origin still represents a major bottleneck due to the slow process of component identification and the scarcity of databases. At present, more and more metabolomic approaches are being applied to search for these metabolites. Accordingly, due to advances entailing the use of high throughput and bioinformatics approaches, metabolomics has become a very useful tool to expand the metabolic profile of agricultural products consumed worldwide, such as peppers, and to investigate the repercussion in human nutrition. In this work, the metabolomic analysis of sweet pepper fruits at two ripening stages and subjected to a NO-enriched atmosphere has been made.

## 2. Results and Discussion

### 2.1. Identification of the Main Metabolites Differentially Detected in Pepper Fruits Depending on the Ripening Stage and the NO Treatment

**Figure 1:**
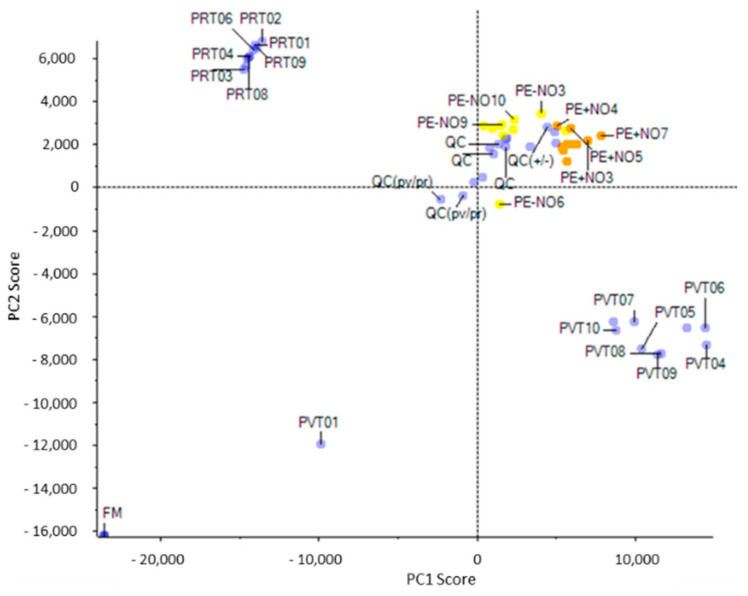
Principal Component Analysis (PCA) of metabolites contained in sweet pepper fruits at different ripening stage and as a consequence of exogenous treatment with NO. A clear grouping of differential metabolites from analysed samples could be observed. The Quality Control (QC) of samples positioned around the same range, indicating their analytical stability. PV, green fruits. PR, red fruits. PE + NO, pepper fruits treated with 5 ppm NO. PE − NO, pepper fruits not treated with NO.

Through the Principal Component Analysis (PCA), the differential presence of metabolites corresponding to each of the groups under study (PV, PR, PE + NO, and PE − NO) was evaluated (Figure 1). Thus, metabolites from green pepper and red pepper were clearly differentiated into two groups (PR vs. PV in the figure). Likewise, groups of differential metabolites were established between fruits not treated (PE − NO) and treated with NO (PE + NO). Additionally, in the PCA, it was observed that the quality control (QC) of samples were grouped, thus validating the analytical system’s performance.

Initially, the number of analytes detected after HPLC-HRMS approaches was 9620. The peaks corresponding to the background noise were eliminated and those corresponding to monoisotopic were selected. Additionally, normalization and groupings were carried out, finally resulting in 2678 analytes. After the differential statistical analysis between the red and green pepper study groups, the result was 1036 differential analytes. The number of metabolites identified differentially in the groups of fruits treated and not treated with NO was 908.

Based on the criteria on the tentative identification previously exposed, the compounds listed in Table 1 were identified thus far.

As observed, colnelenic acid, capsoside A, quercetin and derivatives (quercetin rhamnoside, quercetrin and quercetin 3-(2-Gal-apiosylrobinobioside)), and gingerglycolipid A were more abundant in immature green (PV) than in ripe red fruits (PR), whilst the level of phytosphynosin, FAD, L-tryptophan, tetrahydropentoxylin, blumenol C glucoside and quercetin 3-(3-glucosylrutinoside) was higher in red fruits. Moreover, the NO treatment triggered an elevated content in many of the identified metabolites, including quercetin and derivatives (quercetin rhamnoside, quercetrin and quercetin 3-(2-Gal-apiosylrobinobioside)), gingerglycolipid A, FAD, L-tryptophan, tetrahydropentoxylin, and blumenol C glucoside (PE − NO group). Finally, quercetin 3-(3-glucosylrutinoside) was found to be more abundant in breaking point fruits not treated with NO (PE − NO) (Table 1).

These results convey the feasibility of the experimental design which displays the capacity of discriminating between the different groups set in this study, and can be used to establish accurate metabolic profiles of fruits in different ripening stages as well as in those subjected to modulating molecules such as NO. This strategy could also be followed to investigate the metabolic profile displayed after treatment with other regulatory molecules such as hydrogen sulphide (H_2_S), melatonin, or ethylene, among others. This is quite relevant if we consider the potential nutraceutical effects of some crop products whose metabolic profile may change as has been observed here, with pepper fruits. In previous works, it was found that, for example, ascorbate (vitamin C) content increased due to exogenous treatment of fruits with 5 ppm NO [14], and it also happened in tomato fruits subjected to 300 ppm NO treatment whose content in ascorbate and flavonoids increased by 25% and 60%, respectively [52].

### 2.2. Analysis of the Main Metabolites Differentially Detected in Pepper Fruits through Metabolomics Approaches

#### 2.2.1. Quercetin

**Figure 2:**
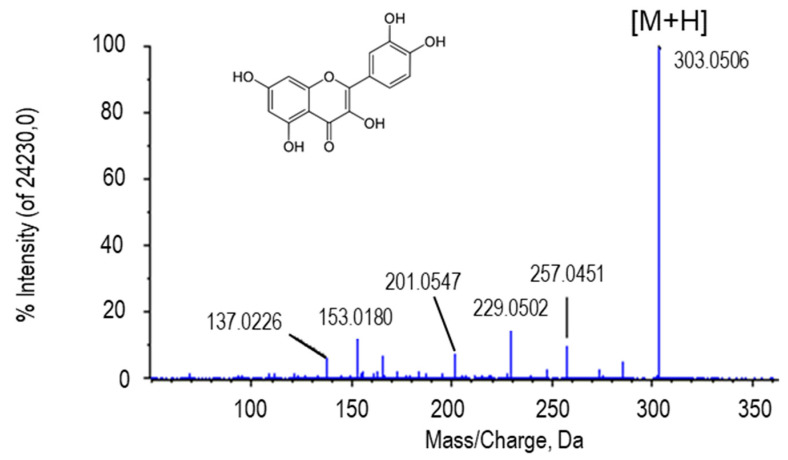
Molecular structure and MS/MS spectrum of quercetin from sweet pepper fruits with the highest intensity peak (303.0506 Da) corresponding to the aglycone form of the metabolite (M) plus one proton [M + H].

Quercetin (Figure 2) is perhaps the main metabolite identified in this work due not only to its repercussion in the plant systems but also to the role this compound and its derivatives play in medicine.

**Figure 3:**
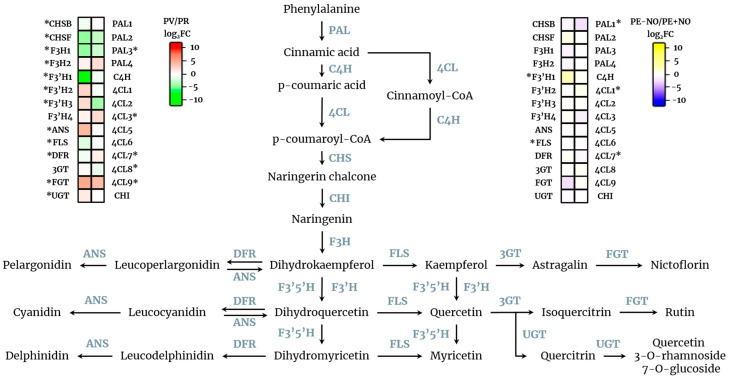
Transcriptomic analysis of the biosynthetic pathway of quercetin and derivatives from sweet pepper fruits. On the left, a heatmap indicating the differentially expressed genes depending on the ripening stage of pepper fruits (red (PR) vs. green (PV)) is depicted with FDR ≤ 0.05. On the right, a heatmap indicating the differentially expressed genes depending on the NO treatment of pepper fruits (treated (PE + NO) vs. untreated (PE − NO)) is depicted with FDR ≤ 0.05. PAL, phenylalanine ammonia-lyase; C4H, trans-cinnamate 4-monooxygenase; 4CL, 4-coumarate-CoA ligase; CHS, chalcone synthase; CHI, chalcone isomerase; F3H, flavanone 3-dioxygenase; F3′5′H, flavanoid 3′,5′-hydroxylase; F3′H, flavonoid 3′-monooxygenase; DFR, dihydroflavonol 4-reductase; ANS, leucocyanidin dioxygenase; FLS, flavonol synthase; 3GT, flavonol 3-o-glucosyltransferase; FGT, flavonol-3-*O*-glucoside L-rhamnosyltransferase; UGT, UDP-glycosyltransferase. Asterisks indicate genes which are differentially expressed in the experimental conditions.

The molecular mass of quercetin is around 302 g/mol, so when a mass spectrum of this metabolite is visualized, a peak of approximately 303 corresponding to this aglycone plus a proton is observed [53]. This peak was detected in the spectra of the four metabolites that were identified as quercetin and quercetin glycosides in our samples (quercetin, quercitrin, quercetin-3-(2Gal-apiosylrobinobioside) and quercetin 3-(3-glucosylrutinoside)) (Figure 3). In addition, depending on the sugar group that each of the quercetin glycosides possesses, a difference was observed between the peaks with the highest intensity of the spectra, corresponding to the indicated loss after fragmentation. The putative identification of this metabolite ((either in the ionized form (+H) and with +Na) was obtained by comparing its fragmentation spectrum with MS/MS obtained from the GNPS databases (results not shown). These compounds derived from quercetin were found to be statistically more abundant in green fruits and after NO treatment (Table 1).

Quercetin is found in a wide variety of fruits and vegetables, including apples, *Brassica*, capers, garlic, grapes, onions, tea, and tomatoes, as well as in many seeds, flowers, barks and leaves from plants and trees, such as *Ginkgo biloba* [54]. Quercetin is about one aglycone, and the presence of a sugar (quercetin glycoside) results in greater water solubility with respect to the aglycone form [54,55]. In plants, quercetin and derivatives have been reported to show antibacterial activity and to contribute to abiotic stress tolerance [56,57]. In lettuce seedlings, it was found that UV-induced quercetin seems to act as phytoanticipins, helping to limit the establishment of biotrophic pathogens by delaying or reducing their sporulation [58]. In addition, it was also detected that a greater induction of the quercetin synthesis was associated with exposure to short UV in *Vicia faba* [59]. Quercetin and derivatives have been already identified in fruits from diverse pepper varieties [8,60,61,62,63]. However, there is still very little data on the quercetin profile and its role throughout the ripening process in pepper fruits [28,64], and nothing is known about its modulation by NO. This work is the first report of the regulatory events which shape the quercetin content in fruits from a higher plant.

The relevance of quercetin as a powerful antioxidant and as a source of other derivative compounds, along with the data shown in this work on its differential content at two ripening stages and under treatment with exogenous NO, provides a new scenario where this flavonoid is perhaps participating in regulatory events associated with fruit physiology. Thus, to elucidate how the quercetin biosynthesis is modulated in pepper fruits under the experimental conditions reported here, an analysis of the expression profile of the genes involved in the synthesis of quercetin and derivatives was made through transcriptomic analysis. As shown in Figure 3 (left panel), many genes were differentially expressed depending on the ripening stage, either green or red. Likewise, the differential expression profile of these genes was also analysed in fruits subjected to NO (right panel). By comparison of the two sides, it is worth noting that genes encoding 4-coumarate-CoA ligase 7 (4-CL7), chalcone synthase F (CHSF), F3′H, flavonoid 3′-monooxygenase 1 (F3′H1), and flavonol synthase (FLS) were regulated by the two assayed conditions, ripening and NO, and perhaps are targets for performing regulatory analyses of this complex pathway in the future.

In humans, quercetin can be taken as a dietary supplement with recommended daily doses of 200–1200 mg [65,66]. Quercetin in food is often presented as quercetin glycoside [67]. As an antioxidant, quercetin has a great capacity to eliminate reactive oxygen species (ROS), such as superoxide radicals (O_2_^−^), and reactive nitrogen species (RNS) such as peroxynitrite (ONOO^−^). This ability of quercetin has been attributed to the presence of two pharmacophores within the molecule that have the optimal configuration to scavenge free radicals. Specifically, it is the catechol group of ring B and the ^−^OH group of C3 [68]. In general, the antioxidant potential of these flavonoids is proportional to the number of free hydroxyl groups [65].

Regarding the bioavailability of quercetin, it has been observed that it is relatively low due to its low absorption, extensive metabolism and rapid elimination [65]. The absorption varies depending on the type of sugar and the conjugation site of these groups [54]. Glycosides are hydrolysed in the small intestine by β-glucosidases to the aglycone form, much of which is then absorbed [65,67]. Studies suggest that quercetin is absorbed in the upper segment of the small intestine [54].

In in vitro assays and tests with some animal models, quercetin has shown a wide range of biological actions including antitumoral, anti-inflammatory and antiviral activities [68]. In addition to its attenuating lipid peroxidation due to its antioxidant activity, quercetin reduces platelet aggregation and capillary permeability, and it also stimulates mitochondrial biogenesis [54]. Among the events that have been observed in vitro, quercetin inhibits the production of enzymes that contribute to inflammation, such as cyclooxygenases and lipoxygenases [66]. Additionally, quercetin blocks inflammation mediated by TNF-α and prevents it from directly activating the extracellular signal-regulated kinase (ERK), the N-terminal c-Jun kinase (JNK), and the nuclear factor κB (NF-κB), which are potent inducers of inflammation. Likewise, quercetin can indirectly prevent inflammation by increasing the activity of peroxisomal proliferator-activated receptors (PPARγ), thus antagonizing the transcriptional activation of inflammatory genes by activating protein 1 (AP-1). Additionally, in adjuvant-induced chronic arthritis in rats, quercetin decreases clinical signs of arthritis compared to untreated controls [54].

The mechanisms of action of quercetin against tumors depend on the type of cancer and its concentration [69]. At high concentrations, it functions as a pro-oxidant and, therefore, can produce chemotherapeutic effects [55]. Most of the anticancer effects are due to the quercetin’s ability to stop the cell cycle by acting on cyclins and cyclin-dependent kinases. It also acts on the PI3K/Akt pathway and the mitogen-activated protein kinase (MAPK) [69]. Moreover, in breast cancer, it has also been observed that quercetin inhibits the production of metalloproteases and causes cell cycle arrest and apoptosis through the regulation of Akt and Bax signaling pathways (proapoptotic proteins of the Bcl family-2) [68].

#### 2.2.2. L-Tryptophan

**Figure 4:**
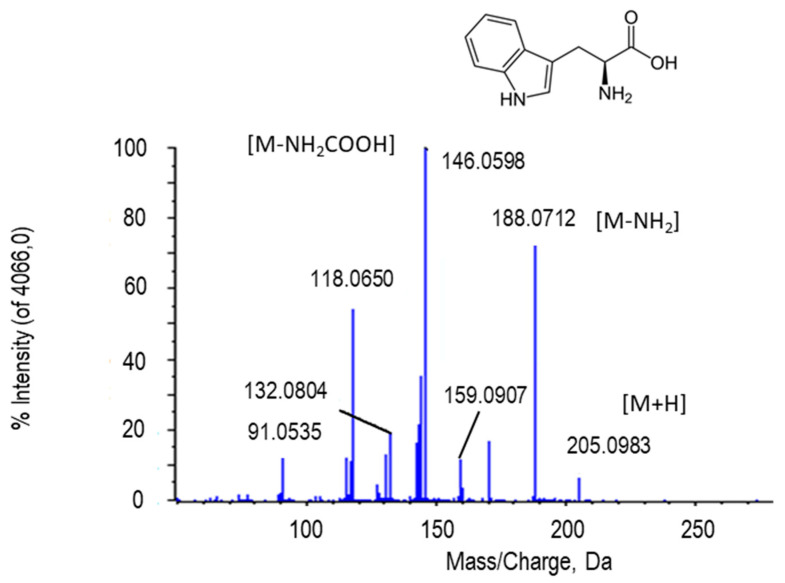
Molecular structure and MS/MS spectrum of L-tryptophan (205.0983 Da) from sweet pepper fruits. The loss of the amino group (-NH_2_) renders the peak at 188.0712 Da, besides the peak (146.0598 Da) corresponding to carboxyl group (-COOH). M, metabolite.

Tryptophan (Figure 4) is an essential amino acid for living beings. In the chromatogram, tryptophan peaked at retention time 3.23 min and *m*/*z* 205.0983 (M + H in the scheme). In the MS/MS spectrum of this metabolite, the peak of 159.0907 Da resulted after the loss of 45 Da, which corresponded to the carboxyl group (-COOH). Moreover, together with the loss of NH_2_, it reached a peak of 146.0598 Da. The peak at *m*/*z* 118.0650 is due to the loss of the side chain (Figure 4). The putative identification of this metabolite was obtained by comparing its fragmentation spectrum with MS/MS from GNPS databases (results not shown).

In humans, tryptophan is obtained from the diet. It is absorbed in the intestine and is metabolized in the colon by the microbiota. In the liver, it is oxidized to acetoacetyl-CoA and is used for the synthesis of NAD^+^. In extrahepatic organs, L-tryptophan is introduced into the kynurenine synthesis pathway. This amino acid and its related metabolites play key roles in various metabolic processes, such as protein biosynthesis and the coordination of the responses of organisms to environmental signals through neurotransmitters and signaling molecules. Imbalances in the amount of tryptophan and related metabolites have been associated with different human pathologies, such as depression, schizophrenia, autoimmunity, and cancer [70].

Indole derivatives from tryptophan catabolism play an important role in key aspects of bacterial physiology and the activation of cells of the immune system. Moreover, tryptophan is the necessary precursor for the synthesis of melatonin in mitochondria (animals and plants), and chloroplasts (plants) where it works as an antioxidant [71]. In fact, melatonin perhaps arose as an ROS scavenger in photosynthetic eukaryotes [72]. In animals, melatonin is involved in the regulation of sleep, the modulation of circadian rhythms, immunity, and as an anti-cancer agent. Likewise, melatonin preserves its ability to reduce oxidative stress through processes that are mostly receptor-independent [73]. Additionally, tryptophan is used in the production of neurotransmitters such as serotonin and neuromodulators like triptamin [70,74].

In plants, it has been reported that tryptophan is the precursor for the phytohormone indole-3-acetic acid (IAA) and the plant defence compounds indole glucosinolates [73,75], and the use of L-tryptophan to improve the productivity of agricultural crops has been also proposed [76]. This is of great interest since the formation of tryptophan-derived compounds in food and edible plants has been highlighted as dietary and metabolic aspects of tryptophan derivatives [77]. In this regard, progress in plant metabolomic engineering for tryptophan overproduction has been made [78].

Since its discovery in 1995, melatonin, as a derivative of the tryptophan catabolism in plants, has gained attention within the scientific community. Thus, it has been described that melatonin in plants promotes seed germination and plant growth and development, and it improves the defence mechanisms and modulates the circadian rhythms [75,79,80,81]. Additionally, melatonin is involved in flowering, fruit setting and ripening [82]. In fact, treatment with melatonin improves the postharvest and storage quality of strawberries and pomegranates [83,84] and confers chilling tolerance to tomato fruits [85].

The results obtained in the present work provide relevance to the research of the tryptophan metabolism in fruits, since, as shown in Table 1, this amino acid is differentially accumulated depending on the ripening stage, with more abundance in ripe red fruits than in immature green ones. Additionally, its promotion through the treatment with NO highlights the cross-talk events which are taking place during ripening, where a fine orchestration of both signalling molecules (NO and melatonin) occurs, as was postulated earlier [86].

#### 2.2.3. Tetrahydropentoxyline

**Figure 5:**
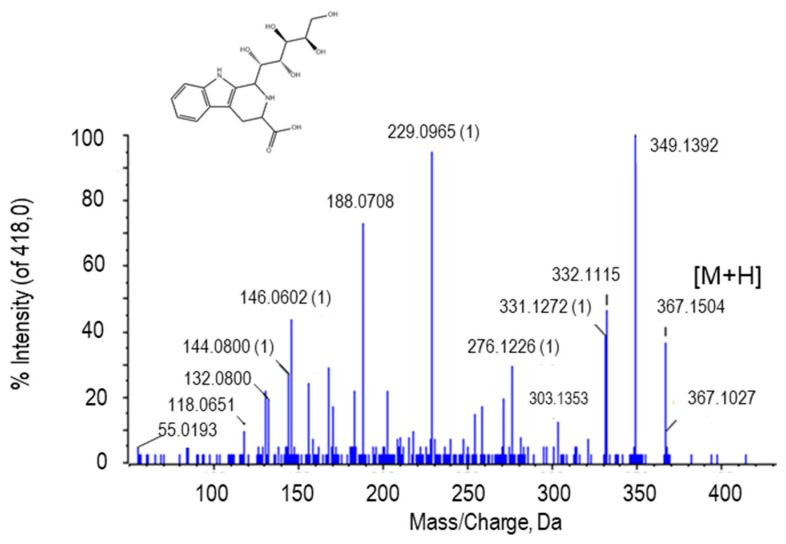
Molecular structure and MS/MS spectrum of tetrahydropentoxyline from sweet pepper fruits. The loss of the 18-Da fragment corresponds to H_2_O group. M, metabolite.

**Figure 6:**
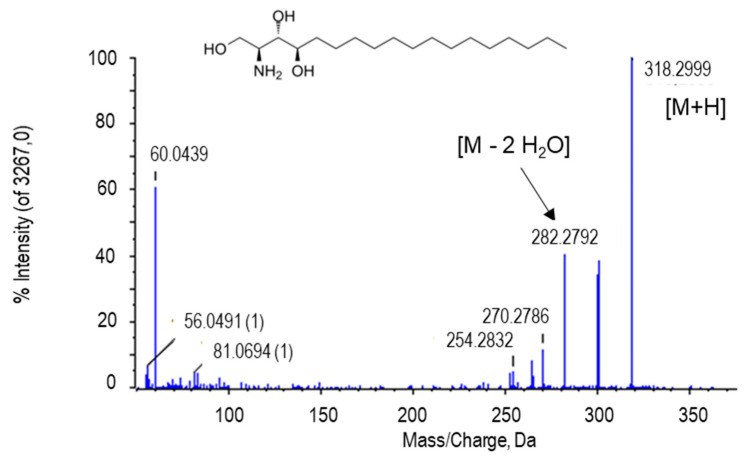
Molecular structure and MS/MS spectrum of the putative phytosphingosine from pepper fruits. M, metabolite.

The peak at *m*/*z* 367.1504 was tentatively identified as tetrahydropentoxyline (THP; Figure 6). THP is a β-carboline produced by the condensation between D-glucose and L-tryptophan. In the MS/MS spectrum of this metabolite, several peaks of loss of the 18 Da could be observed, corresponding to H_2_O groups. The peak at *m*/*z* 229.0965 is due to β-carboline carbosylate containing the alpha methylene group of the side chain (Figure 5). The putative identification of THP was achieved after comparison of its fragmentation spectrum with MS/MS obtained from the GNPS databases.

Tetrahydrocarbolines contain an indole ring that allows them to trap free radicals in a similar way as indole antioxidants [87]. As a matter of fact, in 2015, it was demonstrated that the β-carboline alkaloids were able to scavenge hydroxyl radicals, thus confirming their antioxidant properties [88]. β-carbolines are a great variety of compounds with biological, pharmacological and toxicological effects, whose main activity is based on the inhibition of the monoaminooxidase (MAO), favoring the absorption of monoamines and the binding to benzopyrine and serotonin receptors [88]. These alkaloids have been found in human organs, tissues and fluids due to their intake through diverse foods. This metabolite has been identified in products derived from fruits and vegetables, such as juices, jams, and sauces [89]. Our results report the presence of THP in raw fruits with an abundance profile similar to that of tryptophan, from which it is derived, as indicated above. Its content is also stimulated by NO, which shows the central role played by tryptophan as a precursor of THP and melatonin. The differential pattern of this metabolite in pepper fruits found in this work, with higher contents in ripe red fruits and also promoted by NO, leads us to think that THP may have some role in the ripening process. The connection with NO is still an unreported issue that needs to be investigated further.

### 2.2.4. Phytosphingosine

The peak at *m*/*z* 318.2999 [M + H] and retention time of 8.26 min was tentatively identified as phytosphingosine. The peak at 282.2793 corresponds to the loss of two water molecules (Figure 6). Phytosphingosine is a derivative of sphingosine, also similar to ceramide. However, phytosphingosine has a hydroxyl group in carbon C4 from the sphingoide large chain, whilst sphingosine and ceramide contain a trans double bond between C4=C5 [85]. Phytosphingosine has been reported in microorganisms, yeasts, plants, fungi, and mammal cells as an essential component of the membrane, playing key roles in cell growth, survival and cell death, and mediating the response against stress as secondary messengers [90,91,92,93].

In plants, it has been reported that phytosphingosine participates in plant stress signaling through crosstalk-regulated events [94], and it has been also found that this compound obtained from wheat root exudates displays anti-fungal activity on the rhizosphere microbial community associated with watermelon [95]. In this work, phytosphingosine was more abundant in red fruits than in green ones, indicating that this and other sphingolipids could play a role in the ripening process. On the other hand, our results did not show any phytosphingosine regulation by NO. Nevertheless, it has been reported that the stomatal closure induced by phytosphingosine-1-phosphate depends on NO in guard cells from pea (*Pisum sativum*) [96], and that NO participates in the phosphosphingolipid formation and gene expression triggered by cold conditions [97].

This compound is also found in many mammalian tissues and, interestingly, is associated with some types of cancer cells [90,98]. Phytosphingosine suppresses the ERK1/2 pathway and activates the p38 MAPK pathway with concomitant induction of the translocation of Bax from the cytosol to mitochondria, all of them leading to an intracellular signaling cascade that ends with caspase-dependent cellular apoptosis [99]. In this regard, phytosphingosine was investigated to function as an anticancer agent whose targets include nutrient transport systems and cell vacuolation [100]. Additionally, phytosphingosine stimulates the human keratinocyte differentiation and, in hairless mouse skin, it inhibits the inflammatory epidermal hyperplasia induced by 12-O-tetradeconoylphorbol-13-acetate (TPA) [101]. It has also been found that the interaction of phytosphingosine with the receptor CD300b promotes an NO-dependent neutrophil recruitment induced by zymosan [102].

### 2.2.5. Flavin Adenine Dinucleotide (FAD)

**Figure 7:**
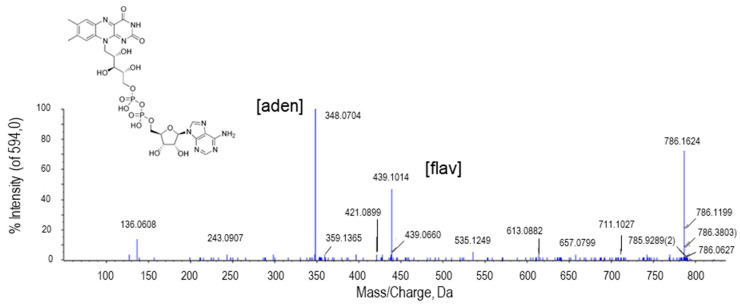
Molecular structure and MS/MS spectrum of FAD from sweet pepper fruits. The peak with the highest intensity (348.0704 Da) corresponds to the adenine nucleotide (aden), whereas that of 430.1014 Da is a consequence of flavin nucleotide (fla).

The peak at *m*/*z* 786.1624 was assigned to flavin adenine dinucleotide (FAD; Figure 7). FAD is one of the nucleotide forms of riboflavin. The breakdown of the FAD by the union of the phosphodiester bond originates two ions, one of them corresponding to the mononucleotide flavin and the other to the nucleotide constituted by the phosphate group, ribose, and adenine. The highest intensity peak (348.0704 Da) corresponded to the adenine nucleotide and the peak of 430.1014 Da to the flavin side (Figure 7).

FAD is the redox cofactor of flavoproteins and participates in reactions that take place in almost all cell compartments, including the mitochondrial electron transport chain, the fatty acid β-oxidation, the pyruvate oxidative decarboxylation, the synthesis of niacin from tryptophan, and the reduction in glutathione by the glutathione reductase, among others. FAD is also necessary for the replication and repair processes of DNA, and its deficiency may lead to a higher risk of DNA mutations [102,103,104,105]. Riboflavin (vitamin B2) is a soluble protein obtained either from food or through the gut microflora. Globally, riboflavin is critical for the good functioning of the metabolism, specifically that associated with nervous, endocrine, cardiovascular and immune systems [106,107,108,109]. In plants, it has been reported that FAD participates in tissue differentiation and organ development [110]. In the present work, FAD content increased in red fruits, indicating that ripening may take place in peppers through metabolic pathways with an enhanced demand of this nucleotide.

### 2.2.6. Other Putative Metabolites

Besides the main compounds reported above, other putative metabolites were identified in pepper fruits, including blumenol C glucoside, colnelenic acid, gingerglycolipid A, and capsoside A (Table 1).

**Figure 8:**
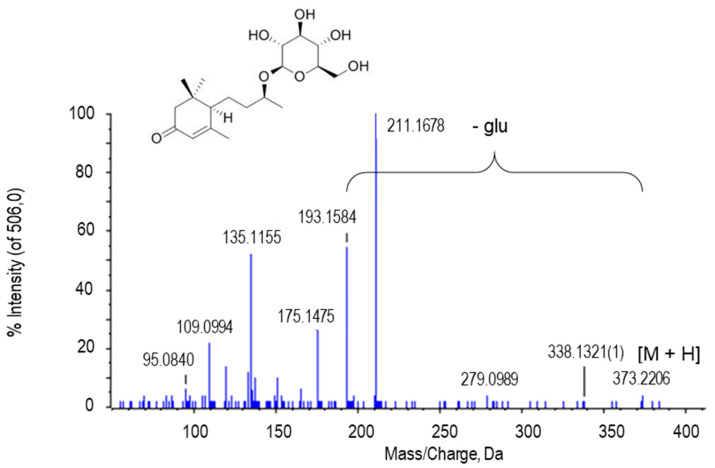
Molecular structure and MS/MS spectrum of blumenol C glucoside from sweet pepper fruits. The peak with 193.1584 Da is generated after the loss of the glucose (glu) residue. M, metabolite.

Blumenol C glucoside was tentatively assigned to the peak at *m*/*z* 373.2206. The peak obtained from the blumenol C glucoside that was registered in the MS/MS spectrum at 193.1584 corresponded to the loss of glucose (Figure 8).

This is a fatty monoacyl glycoside that has been identified in plants from the *Solanaceae* family, specifically in tobacco. This type of molecule is associated with arbuscular mycorrhizal fungi (AMF), which establish symbiosis with plants to facilitate the absorption of phosphorus and nitrogen. Furthermore, some blumenol compounds have been found in plant families that have lost their ability to establish AMF interactions, such as *Brassicaceae* [111]. On the other hand, in a study with strawberry metabolites, it was observed that blumenol C glucoside has cytotoxicity against the human nasopharyngeal carcinoma cell line [112]. This subject is a candidate for investigation since no data of the presence on this compound in pepper have been published thus far. Additionally, no reports associated with fruit ripening and interaction with NO have been provided yet.

**Figure 9:**
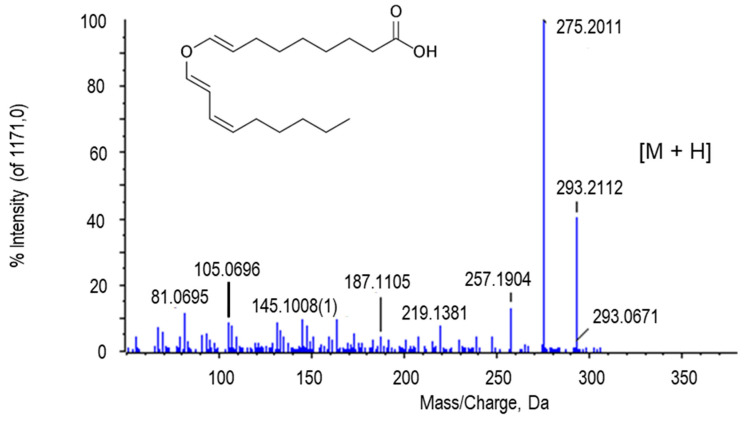
Molecular structure and MS/MS spectrum of colnelenic acid from sweet pepper fruits. The peak with the highest intensity (275.2011 Da) was obtained after a decrease of 18 Da as consequence of a H_2_O loss. M, metabolite.

The peak of *m*/*z* 293.2112 with the retention time of 10.69 min was presumably assigned to colnelenic acid (Figure 9). This metabolite belongs to the group of oxylipins, oxygenated fatty acids that are generated in plant cells during development and against environmental stress and attack by pathogens [113,114].

Oxylipins have also been associated with cancer therapies [115]. Colnelenic acid is produced through an enzymatic pathway mediated by lipoxygenase whose precursor is linoleic acid [116]. In pepper fruits, the isoenzymatic lipoxygenase profile was recently reported after non-denaturing PAGE, and only one isozyme was detected. The same study did not show any difference in the lipoxygenase activity due to either fruit ripening or treatment with NO [42], although in this work, colnelenic acid was more abundant in green fruits. This subject deserves to be further investigated in pepper fruits since, to our knowledge, there is very little information on oxylipins and lipoxygenase metabolism in pepper.

**Figure 10:**
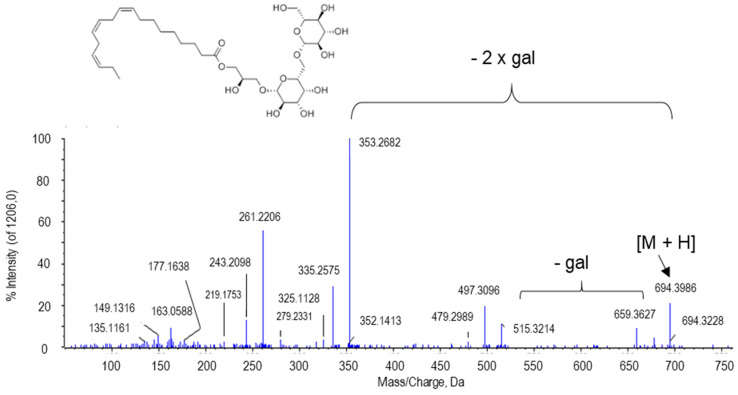
Molecular structure and MS/MS spectrum of gingeglycolipid A [M + NH_4_] from sweet pepper fruits. The peak at 515.3214 Da corresponds to the ion resulting from the loss of the first galactose (gal), and the peak at 335.2575 was generated after the second galactose is lost. M, metabolite.

The peak at *m*/*z* 694.3986 was presumably identified as gingerglycolipid A. Two typical losses of gingerglycolipid A after ionization and QTOF fragmentation are those involving two galactose residues. Thus, in the MS/MS spectrum of this metabolite, the peak at 515.3214 Da was identified, corresponding to the ion resulting after the first galactose, whereas the peak at 335.2575 Da resulted after the second galactose was lost (Figure 10).

Gingerglycolipid A is one of the three monoacyldigalactosyl glycerols that have been identified in the rhizome of *Zingiberis*, commonly referred to as ginger [117], although it has also been reported in leaves of the Brazilian folk medicinal plant *Sideroxylon obtusifolium* [118]. Ginger is a plant that has medicinal properties, as it was confirmed that it has an antiemetic effect, it is effective against arthritic diseases, and against tumor growth, rheumatism, and migraine [117,119]. To our knowledge, this is the first report of the presence of gingerglycolipid A in fruits from a higher plant. As shown in Table 1, gingerglycolipid A seems to be differentially accumulated depending on the ripening stage and the treatment with NO. This makes this molecule a key point for expanding the investigation of the pepper fruit metabolome and its therapeutic uses, since they might be potentiated by NO.

**Figure 11:**
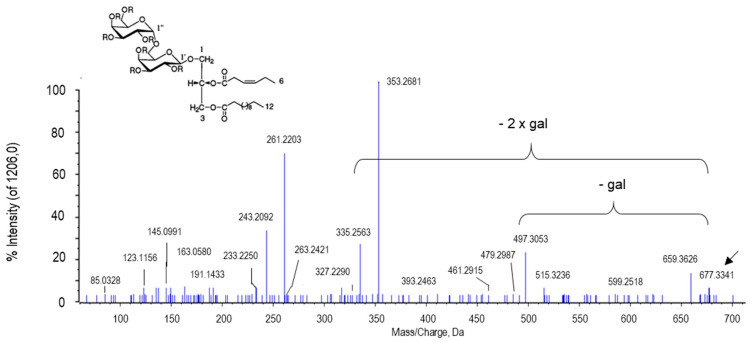
Molecular structure and MS/MS spectrum of capsoside A from sweet pepper fruits. The peak at 497.3096 Da corresponds to the ion resulting from the loss of the first galactose (gal), and the peak at 353.2682 was generated after the second galactose was lost.

The peak of *m*/*z* 677.3341 with the retention time of 9.54 min was tentatively assigned to capsoside A (Figure 11). Capsoside A is also a diacyldigalactosyl glycerol like gingerglycolipid A. In fact, the difference of the mass/charge ratio between both compounds resides in a water group. In the MS/MS spectrum of capsoside A, a major peak at 497.3053 was also obtained that corresponded to the loss of one galactose residue. Some of the most apparent peaks observed for gingerglycolipid A (694.3986, 515.3214, 353.2682 and 335.2575) and for capsoside A (677.3341, 497.3053, 353.2681 and 335.2563) were obtained after the lost/gain of 1–2 galactoses and 1 H_2_O group. Capsoside A has been exclusively identified from pepper fruits in 2001 along with capsoside B, capsianoside VII and other glycosides. This chemical was found to be more relevant at the green stage of pepper fruits, where the activation of β-galactosidase takes place, releasing free galactose from the membrane and playing an important role in the fruit ripening [120,121]. This behavior agrees with our data which detected that capsoside A was more abundant in green fruits.

## 3. Materials and Methods

### 3.1. Plant Material

**Figure 12:**
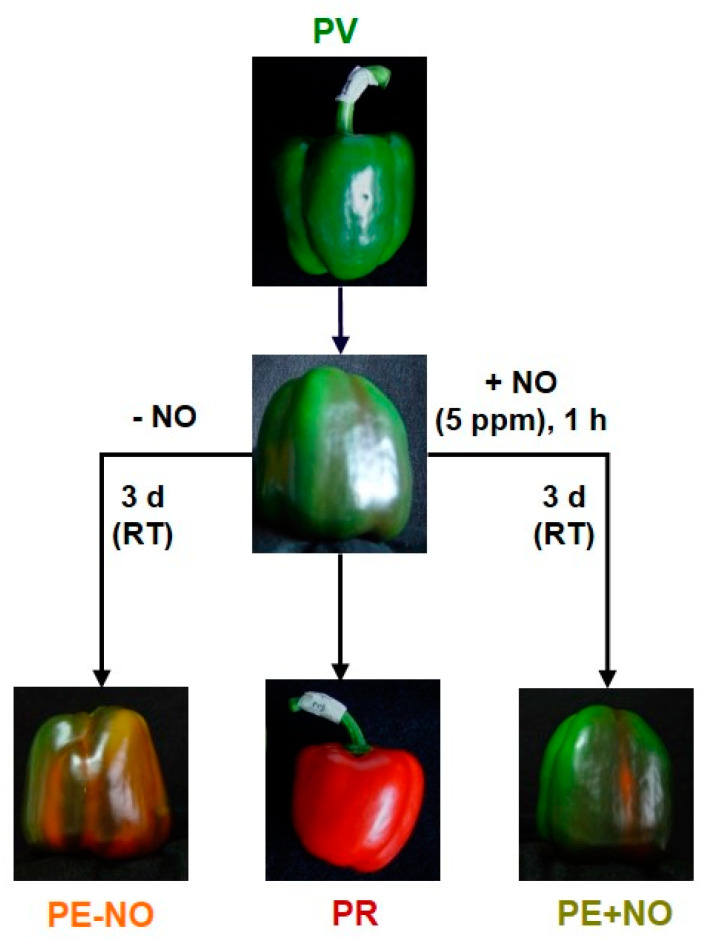
Experimental design to set four groups of pepper fruits for metabolomic analysis. PV, immature green. PR, ripe red, PE + NO, breaking point fruits incubated with 5 ppm NO for 1 h and further storage for 3 d at room temperature (RT). PE − NO, breaking point fruits not treated with NO for 1 h and further storage for 3 d at RT. (Reproduced with permission from González-Gordo et al. [122]).

Pepper fruits (*Capsicum annuum* L.) of the California sweet type, Melchor variety, were provided by the Syngenta Seeds Ltd./Zeraim Iberica (El Ejido/Roquetas de Mar, Almería, Spain). For our study, four groups of pepper fruits were used according to the experimental design reported earlier [50,51,122,123]: immature green (PV), ripe red (PR), breaking point treated with NO (PE + NO) and breaking point without NO treatment (PE − NO). Green and red fruits were collected from plants grown in experimental greenhouses at the Syngenta Seeds/Zeraim Iberica facilities. For NO treatment, breaking point fruits were incubated in the presence of 5 ppm NO in hermetic boxes for 1 h. Then, fruits were maintained for 3 days at room temperature. A parallel group of breaking point fruits without NO treatment was run at the same time [122,123] (Figure 12).

### 3.2. Preparation of Fruit Extracts

Samples of these four types of fruits were collected and washed with distilled water. Subsequently, they were dried, chopped into cubes of about 5 mm each edge, and treated with liquid nitrogen to store the samples at −80 °C until their use. For each of the pepper groups, ten samples were taken and were powdered under liquid N_2_ using an electronic grinder IKA^®^ A 11 BASIC (IKA Laboratories Inc., Tirat Carmel, Israel). Then, 1 g of each sample was dissolved into 1 mL acetonitrile (AcN, analytical grade). The extracts were shaken for two minutes with a multi-vortex at maximum speed, and subsequently centrifuged for 15 min at 16,000 g and 4 °C. Supernatants were collected and evaporated with a nitrogen gas stream. Then, 100 µL of 20% (*v/v*) dimethylsulfoxide (DMSO), and 100 µL of 50% (*v*/*v*) AcN in ultrapure water were added to each extract.

### 3.3. High-Performance Liquid Chromatography Coupled to High Resolution Mass Spectrometry (HPLC-HRMS)

The chromatographic separation technique was carried out using an Agilent Series 1290 equipment. The column used was an Atlantis T3 type (C18: 2.1 mm × 150 mm, 3 µm) (Waters Corporation, Milford, MA, USA)) maintained at 25 °C. The elution method followed was gradient-type, with 5 µL of each sample being injected and the flow used was 0.3 mL/min. Eluent A corresponding to the mobile phase consisted of 0.1% (*v*/*v*) formic acid in 90:10 AcN:water. Eluent B was a mixture of 0.1% (*v*/*v*) formic acid in 90:10 water:AcN. The chromatographic run was 20 min with gradient elution mode of mobile phases A/B as follows: 0–0.5 min 1% eluent B, 0.5–11.0 min 99% B, 11.0–15.5 min 99% B, 15.5–15.6 min 1% B and 15.6–20.0 min 1% B.

The mass spectrometer used for detecting the metabolites was a Triple TOF 5600 quadrupole-time of flight (SCIEX, Framingham, MA, USA). The source temperature at the set point was 500.0 °C with a DuoSpray Ion Source (SCIEX), and the pressure was around 193 bar. To carry out the detection, a positive electrospray ionization mode (ESI +) was used, and the fragmentation mode was information-dependent acquisition (IDA) with a mass tolerance range of 50 mDa.

### 3.4. Analysis of Metabolomics Data

The data obtained after HPLC-MS were processed using Marker View software version 1.2.1 (SCIEX; https://sciex.com/products/software/markerview-software, accessed on 24 April 2021). From the data, a matrix was generated where each row corresponded to an ion with its corresponding retention time and a specific mass/charge (*m*/*z*) ratio. Retention times were aligned to correct for the possible drift of the same ion between samples. Next, the *t*-Student test was applied with a confidence level of 95% (*p* < 0.05) to determine the differential mass signals between the mobile phase and the samples under study.

Subsequently, the statistical analysis was carried out using the Metaboanalyst 4.0 Web Server (University of Alberta, Edmonton, AB, Canada, https://www.metaboanalyst.ca, accessed on 24 April 2021) platform. This analysis consisted of performing the normalization, scaling, and transformation of the data obtained, to reduce the variations between the measurements of the samples. Through a principal component analysis (PCA), the performance of the experimental and analytical system was observed by evaluating the QC (quality control).

A statistical analysis was performed using *t*-Student (*p*-value < 0.05) to detect the differential analytes between samples from all four groups of pepper fruits.

### 3.5. Metabolomic Identification

The tentative identification of metabolites was carried out based on three criteria: (i) an exact mass error below 5 ppm; (ii) the equivalence of the isotopic profile with a variation in the intensity ratio of less than 20%; and (iii) matching the fragmentation spectrum with databases.

Initially, using the MarkerView platform, the chromatographic signals, the spectrum, and the extracted-ion chromatogram (XIC) of each selected analyte were checked, as well as the possible existence of its MS/MS spectrum. Then, in the PeakView software (SCIEX, https://sciex.com/products/software/peakview-software, accessed on 24 April 2021), a new XIC session was opened for the analysis of the relevant sample, selecting each metabolite by its corresponding *m*/*z* value and its retention time. The Formula Finder tool (PeakView software) was used to estimate molecular formulas based on the MS and MS/MS spectra. The files from MarkerView were also uploaded to the NIST 2012 database (mass spectral library) (National Institute of Standards and Technology, Gaithersburg, MD, USA, https://www.nist.gov/itl/iad/image-group/fpvte-2012, accessed on 24 April 2021).

Furthermore, the identification was supported using several databases indicating the experimental mass, tolerance (maximum error in ppm), *m*/*z*, ionization mode, and adducts. To reduce the number of possible metabolites, the fragmentation data was checked with the MS/MS libraries: MassBank (Mass Spectrometry Society of Japan, Yamabuki-cho, Shinjuku-ku, Tokyo, Japan, www.MassBank.jp/, accessed on 24 April 2021); METLIN (La Jolla, CA, USA, metlin.scripps.edu/index.php, accessed on 24 April 2021); GNPS (University of California, San Diego, CA, USA, gnps.ucsd.edu, accessed on 24 April 2021); and ReSpect database (spectra.psc.riken.jp, accessed on 24 April 2021), which is a plant-specific MS/MS database compiled by the RIKEN Institute (Hirosawa, Wako, Saitama, Japan). The Human Metabolism Database (HMDB) (The Metabolomic Innovation Center, Edmonton, AB, Canada, www.hmdb.ca/, accessed on 24 April 2021) provides coverage of 41,993 metabolites and 5774 experimental MS/MS, and 27,999 predicted in silico MS/MS spectra. Ceu Mass Mediator, Universidad CEU-San Pablo, Boadilla del Monte, Madrid, Spain, http://ceumass.eps.uspceu.es/, accessed on 24 April 2021) is a database that encompasses integrated real compounds from various metabolic pathways and includes 279,318 compounds. MAGMa (Netherlands Metabolomics Center, Wageningen, The Netherlands, http://www.emetabolomics.org/magma, accessed on 24 April 2021) is a metabolomics tool that allows comparing experimental MS/MS spectra with metabolites described in the PubChem (National Center for Biotechnology Information, Bethesda, MD, USA, https://www.ncbi.nlm.nih.gov/, accessed on 24 April 2021), KEGG (Kyoto Encyclopedia of Genes and Genomes, Kanehisa Laboratories, Kyoto, Japan, https://www.genome.jp/kegg/, accessed on 24 April 2021) or HMDB databases, or importing the target molecule structure. The PeakView tool of association of a structure to a certain MS/MS spectrum was also used, which works similarly to MAGMa and allows selection of regions of the structure to check their molecular weight.

Based on the results obtained throughout the workflow, the metabolites described in plants were selected with the help of the Dictionary of Natural Products program (CRC Press, Taylor & Francis Group, Boca Raton, FA, USA, http://dnp.chemnetbase.com/faces/chemical/ChemicalSearch.xhtml, accessed on 24 April 2021).

### 3.6. Transcriptomic Analysis

Routine protocols, platform, programs and bioinformatics tools for RNA extraction from samples, library preparation, sequencing, assembling sequences and annotation of the pepper fruit transcriptome were those reported in detail previously. Basically, libraries were sequenced on an Illumina NextSeq550 system (Illumina, Inc., San Diego, CA, USA) using four independent replicates from green fruits (PV) and five belonging to groups PR, PE + NO and PE − NO (Figure 12) [51,122]. Raw sequence reads were deposited in the Sequence Read Archive (SRA) of the NCBI and are accessible through accession number PRJNA668052 (National Center for Biotechnology Information, Bethesda, MD, USA, https://www.ncbi.nlm.nih.gov/sra/PRJNA668052, accessed on 24 April 2021).

Differential expression analyses were performed using DEgenes-Hunter [124] with default parameters to examine the relative change in expression among the ripening stages (PV vs. PR) and the NO treatment (PE − NO vs. PE + NO). A false discovery rate (FDR) of 0.05 was used for identifying differentially expressed genes (DEGs). Protein identifiers corresponding to the biosynthetic pathway of quercetin and its derivatives were obtained using KEGG and UniProt (UniProt Consortium, Geneva, Switzerland, https://www.uniprot.org/, accessed on 24 April 2021) databases and were compared against our datasets. The mean of the log2 fold change (log_2_FC) obtained with EdgeR [125], DESeq2 [126], Limma [127], and NOISeq [128], was used to generate the heatmaps of the corresponding genes.

## 4. Conclusions and Future Prospects

Based on the results obtained in this work, it could be considered that pepper has a great variety of bioactive compounds at both maturation stages. Fruits and vegetables provide a preventive value against several disorders, for example, cancer and cardiovascular diseases, due, among other compounds, to antioxidants such as vitamins A, C and E, carotenoids and polyphenols or flavonoids. These compounds can protect the body against both ROS and RNS involved in most disease states.

It should be noted that the metabolic profile reported in this work was found to be influenced by the ripening stage and the treatment with NO. This is quite interesting since it allows designing a strategy to obtain the optimal content of specific metabolites for their further pharmacological and therapeutic uses. The pattern followed by quercetin and its derivatives (quercitrin, quercetin rhamnoside, quercetin 3-(2Gal-apiosylrobinobioside)) and by gingerglycolipid A should be highlighted since these metabolites are found more abundantly in green fruits and in those treated with NO. These results were also corroborated by expression analysis of the genes involved in the quercitin synthesis carried out by transcriptomic approaches. The content of L-tryptophan, which is the precursor of melatonin and serotonin, was also favored by NO. In the last decade, it was demonstrated that in sweet pepper fruits, NO delays fruit ripening, with NO-treated fruits somehow resembling the metabolic profile observed in green fruits [38]. Later, it was proved that NO stimulated the gene expression and enzyme activity of galactono-1,4-lactone dehydrogenase, the last enzyme of the ascorbate biosynthesis, and these activating episodes were accompanied by an enhancement of ascorbate content of up to 40% [14]. A similar trend was also reported in tomato fruits subjected to exogenous NO [53]. Therefore, considering the roles played by ascorbate and quercetin at therapeutic levels, it can be hypothesized that treatment with NO provokes an enrichment of beneficial molecules in pepper fruits, and this can be used for future strategies conducted to obtain fruits with added value. Likewise, the present results invite us to think that we can set the best ripening moment with the highest values of certain molecules with pharmacological potentiality.

In this work, previously unreported metabolites in pepper fruits have been identified (tetrahydropentoxyline, acid colnelenic, blumenol C glucoside, and gingerglycolipid A), and they have also been found to be modulated by the ripening process and by NO. These, and the above mentioned findings, open new perspectives for the investigation of the entire pepper metabolome and how to manage it in order to consider this plant species more than a food or condiment. The great versatility of pepper fruits in terms of culinary uses, nutritional value, and the considerably increasing consumption and production in the last years make this crop a central player, as a nutraceutical vector which might contribute to improving our health status through the daily consumption of compounds with therapeutic value. Consequently, biotechnological strategies could be applied for optimizing the level of these beneficial compounds. In addition to this view, this investigation is also a starting point for initiating new studies on the specific function of these metabolites in the physiology of sweet pepper and other edible fruits.

## Figures and Tables

**Table 1:** Metabolites tentatively identified in samples of red and green sweet pepper fruits, and treated or not with nitric oxide (NO). The name of the compound, its molecular formula, the mass/charge value (*m*/*z*) and the retention time obtained in the metabolomic analysis are indicated. The associated adduct with the metabolite is also recorded, as well as its ppm error. In addition, the state of maturation (PV, green/PR, red) and treatment of the fruit (PE + NO, treated with NO/PE − NO, not treated with NO) in which each metabolite was found differentially more abundant are indicated, together with the corresponding *p*-values.

Metabolite	Formula	*m*/*z*	Retention Time	Aduct	Error ppm	Ripening Stage/ +/− NO	*p*-Value
Colnelenic acid	C_18_H_28_O_3_	293.2121	10.69	H^+^	3	PV	6.72 × 10^−12^
Capsoside A	C_33_H_58_O_15_	677.3754	9.54	-H_2_O^+^/H^+^	0	PV	1.049 × 10^−7^
Quercetin	C_15_H_10_O_7_	303.0507	5.40	H^+^	2	PV/ PE+NO	1.68 × 10^−13^/ 1.13 × 10^−16^
Quercitrin (Quercetin rhamnoside)	C_21_H_20_O_11_	449.1074	5.38	H^+^	0	PV/ PE+NO	2.278 × 10^−13^/ 3.92 × 10^−18^
Quercitrin	C_21_H_20_O_11_	471.0892	5.40	Na^+^	0	PV/ PE+NO	3.65 × 10^−16^/ 5.47 × 10^−14^
Gingerglycolipid A	C_33_H_56_O_14_	694.4012	9.51	NH_4_^+^	0	PV/ PE+NO	1.402 × 10^−8^/ 0.025
Quercetin 3-(2Gal-apiosylrobinobioside)	C_32_H_38_O_20_	743.2052	3.74	H^+^	3	PV/ PE+NO	7.396 × 10^−7^/ 2.454 × 10^−9^
Phytosphingosin	C_18_H_39_O_3_	318.3004	8.26	H^+^	0	PR	1.34 × 10^−10^
FAD	C_27_H_33_N_9_O_15_P_2_	786.1624	4.97	H_+_	2	PR	1.453 × 10^−12^
L-Tryptophan	C_11_H_12_N_2_O_2_	205.0959	3.23	H^+^	3	PR/ PE+NO	8.01 × 10^−14^/ 3.53 × 10^−5^
Tetrahydropentoxylin	C_17_H_22_N_2_O_7_	367.1509	3.01	H^+^	2	PR/ PE+NO	5.21 × 10^−5^/ 0.0492
Blumenol C glucoside	C_19_H_32_O_7_	373.2216	5.35	H^+^	1	PR/ PE+NO	1.87 × 10^−10^/ 0.00189
Quercetin 3-(3-glucosylrutinoside)	C_33_H_40_O_21_	773.2133	3.69	H^+^	0	PR/ PE-NO	1.14 × 10^−10^/ 1.42 × 10^−7^

## Data Availability

Raw sequence reads from sweet pepper fruits were deposited in the Sequence Read Archive (SRA) of the NCBI and are accessible through accession number PRJNA668052 (National Center for Bio-technology Information, Bethesda, MD, USA, https://www.ncbi.nlm.nih.gov/sra/PRJNA668052, accessed on 24 April 2021).
